# Analysis of Contact Traces of Cannabis by In-Tube Solid-Phase Microextraction Coupled to Nanoliquid Chromatography

**DOI:** 10.3390/molecules23092359

**Published:** 2018-09-15

**Authors:** Neus Jornet-Martínez, Adrián Ortega-Sierra, Jorge Verdú-Andrés, Rosa Herráez-Hernández, Pilar Campíns-Falcó

**Affiliations:** MINTOTA Research Group, Department of Analytical Chemistry, Faculty of Chemistry, University of Valencia, Dr. Moliner 50, 46100 Burjassot, Valencia, Spain; neus.jornet@uv.es (N.J.-M.); aorsie@alumni.uv.es (A.O.-S.); jorge.verdu@uv.es (J.V.-A.); pilar.campins@uv.es (P.C.-F.)

**Keywords:** in-tube solid-phase microextraction (IT-SPME), nanoliquid chromatography (nanoLC), contact trace analysis, cannabis, THC

## Abstract

Because of its inherent qualities, in-tube solid-phase microextraction (IT-SPME) coupled on-line to nanoliquid chromatography (nanoLC) can be a very powerful tool to address the new challenges of analytical laboratories such as the analysis of traces of complex samples. This is the case of the detection of contact traces of drugs, especially cannabis. The main difficulties encountered in the analysis of traces of cannabis plants on surfaces are the low amount of sample available (typically < 1 mg), the complexity of the matrix, and the low percentages of cannabinoic compounds in the samples. In this work, a procedure is described for the detection of residues of cannabis on different surfaces based on the responses obtained by IT-SPME coupled to nanoLC with UV diode array detection (DAD) for the cannabinoids Δ^9^-tetrahydrocannabinol (THC), cannabidiol (CBD), and cannabinol (CBN); the proposed conditions can also be applied for quantitative purposes through the measurement of the percentage of THC, the most abundant cannabinoid in plants. The method is based on collecting the suspected drug samples with cotton swabs, followed by the extraction of the target compounds by ultrasound assisted extraction. The extracts are then separated and processed by IT-SPME-nanoLC. The proposed approach has been applied to the detection of traces of cannabis in different kind of items (plastic bags, office paper, aluminum foil, cotton cloths, and hand skin). Sample amounts as low as 0.08 mg have been collected and analysed for THC. The selectivity and effect of the storage conditions on the levels of THC have also been evaluated. The percentages of THC in the samples typically ranged from 0.6% to 2.8%, which means that amounts of this compound as low as 1–2 µg were adequately detected and quantified. For the first time, the reliability of IT-SPME-nanoLC for the analysis of complex matrices such as cannabis plant extracts has been demonstrated.

## 1. Introduction

The detection and characterization of contact traces of some substances, such as illicit drugs or explosives, is a challenging task that needs to be addressed in some investigations. For example, the presence of drug traces on clothes, packaging, skin, or vehicle interiors may be due to simple contact with the bulk drug during its production, transport, or consumption and may persist for relatively long periods of time. Thus, positive identification and characterization of drugs in these kinds of samples may play a significant role in criminal investigations, particularly in those related to drug trafficking [1,2,3]. The main difficulty in the analysis of contact traces of drugs is the low amount of available sample (<1 mg) that is often only visible through microscopy. Furthermore, unlike other illicit drug samples (e.g., amphetamine-derived drug street samples), a large number of constituents are present in cannabis plants, and the percentages of the cannabinoic compounds in them are usually low. Cannabis is one of the most important products in the illicit drug market. For example, in Spain, although the private possession of small amounts of cannabis for consumption is allowed, the lucrative sale of this product is illegal and it is the predominant material seized in the context of drug trafficking control activities [4]. Therefore, high sensitivity and selective techniques for the analysis of traces of cannabis are required.

Some spectroscopic techniques have been proposed for the direct detection of residues of drugs on different items. For example, the detection of traces of cocaine on dealers’ clothes and cars has been reported using ion mobility spectrometry [1]; Raman spectroscopy and ambient mass spectrometry have been applied to detect cocaine, amphetamine, ketamine, and *N*-methyl-3,4-methylenedioxy methamphetamine (MDMA) in a variety of fabrics [5,6,7]. These techniques have proved to be useful for samples with few components, mostly one or two active drugs and some adulterants. However, for the analysis of more complex samples such as cannabis, chromatographic techniques are predominant because the psychoactive ingredients used for identification and quantification have to be separated from the other plant constituents. The samples are generally collected with swabs or wipes that are subsequently treated to extract the target compounds. Finally, the extracts are analysed, typically by liquid chromatography (LC) coupled on-line to mass spectrometry (MS). This has been the approach used for the analysis of traces of cannabis on police station work surfaces as well as in the clothes and hands of workers involved in the custody and/or destruction of drug seizures for assessing the levels of exposure to THC [8,9,10,11]. Multiple extraction and recombination of the extracts followed by solvent evaporation is necessary in order to reach the required sensitivity.

The miniaturization of the chromatographic systems (capillary LC and nanoLC) is one of the options available to improve the sensitivity of chromatographic analysis [12]. The sensitivity can be further increased if an on-line preconcentration technique such as in-tube solid-phase microextraction (IT-SPME) is used [13,14]. In this form of microextraction, the analytes are concentrated in an extractive capillary packed or coated with a proper sorbent. Although there are different modalities to achieve the extraction, the employment of an extractive capillary as the loop of the injection valve of the liquid chromatograph (in-valve IT-SPME) is one of the most attractive options for organic analytes. This is because the target compounds are retained in the capillary during sample loading and sent to the analytical column with the mobile phase when changing the valve position. In such a way, relatively large volumes of the sample can be loaded into the system until the required amount of the target compound is retained in the capillary. The potential of IT-SPME coupled to capillary LC has been extensively documented for a variety of organic compounds [13,14,15], and more recently, IT-SPME coupled to nanoLC has been applied to the analysis of some pollutants in water samples [16]. 

In the present work, we describe a new method for the detection and quantification of contact traces of cannabis in different kinds of surfaces using in-valve IT-SPME coupled on-line to nanoLC. The method is based on the employment of cotton swabs for collecting the suspected sample, the extraction of the cannabinoids by treating the cotton swabs in an ultrasonic bath, and the direct processing of the extracts by IT-SPEM-nanoLC. Cotton swabs were selected for sampling, as it is the methodology commonly used to collect residues of drugs from surfaces [8,9,10,11]. Δ^9^-tetrahydrocannabinol (THC), cannabidiol (CBD), and cannabinol (CBN) were the target analytes selected; for quantitative purposes, THC was the only compound used because it was the predominant cannabinoid in plants. The IT-SPME and chromatographic conditions were previously optimized using extracts obtained from cannabis plants. The proposed approach has been successfully used for the detection of residues of cannabis plants (<1 mg). The selectivity towards other plants (infusion herbs, tobacco) and the effects of storage conditions and item type have been studied.

## 2. Results

### 2.1. Chromatographic Analysis

Conditions used to effect IT-SPME of the target cannabinoids were selected according to the results obtained in our previous works. A polydimethylsiloxane-based coated capillary (TRB5) was used because this phase provided satisfactory results in the extraction of compounds of similar polarity [15]; the sample volume and the capillary length were selected taking into account the dimensions of the chromatographic column and the mobile-phase flowrate [16]. Initially, different experiments were carried out in order to find chromatographic conditions suitable for the separation of THC, CBD, and CBN from other plant constituents. The selected elution conditions allowed a satisfactory separation of CBD (retention time, t_r_ = 13.3 min), CBN (t_r_ = 15.1 min), and THC (t_r_ = 16.2 min) from the rest of the components extracted from the plants, as most of them were expected to elute at shorter retention times [17,18,19]. 

The quantitative performance of IT-SPME-nanoLC was studied by processing aqueous solutions of the analytes. The results obtained are listed in Table 1. As it can be observed, satisfactory linearity was found for the three compounds studied up to concentrations of 100 ng/mL. The instrumental limits of detection (LODs), established for each analyte as the concentration that provided a signal-to-noise ratio (S/N) of 3, were 2 ng/mL for THC and 5 ng/mL for the other compounds, and the limits of quantification (LOQs) (established for an S/N of 10) were 8–15 ng/mL. 

The analytical performance of the proposed conditions was considered satisfactory and, therefore, applied to evaluate the presence and concentrations of THC, CBD, and CBN in the extracts obtained from cannabis plants. 

### 2.2. Analysis of Extracts of Cannabis Plants

Four cannabis samples (M1–M4) were used throughout the study. These samples were analysed in order to estimate the content in cannabinoids for subsequent comparison with the results of the studies with residues. Samples were roughly homogenized manually, and portions of 10 mg were subjected to extraction with 3 mL of a mixture of methanol and chloroform (9:1 (*v*/*v*)). Extractions were performed in an ultrasonic bath for 15 min as proposed in [16]. The liquid phase was then separated and filtrated (<0.20 µm), and portions of 10 µL were analysed by IT-SPME-nanoLC. As the amounts of cannabinoids vary with the storage conditions, portions of the samples that were dried at 50 °C for 6 days were also analysed.

The direct injection of the collected extracts saturated the detector, particularly at retention times of 9–13 min, where most matrix components eluted. Moreover, the peak areas obtained for THC were much higher than those obtained for standards at concentrations within the linear range (see Table 1). It has to be noted that for in-valve IT-SPME and for a given analyte and extractive phase, the extraction efficiency is mainly determined by the solvent sample composition [13,14]. For the extraction of low-medium polarity compounds, such as the cannabinoids used in the present study with apolar coatings (such as TRB 5), the analytes must be loaded in the capillary in a water-rich eluent [20], otherwise, they do not interact with the extractive coating and are excluded from the capillary during sample loading. For these reasons, in the present study, the extracts obtained from the plants were diluted with ultrapure water before being processed by IT-SPME. 

A dilution factor of 1:100 led to suitable chromatographic profiles and adequate peak areas for the predominant cannabinoid (THC). It was observed that the peak of THC increased after drying at 50 °C, most probably due to the loss of humidity. In all the samples assayed, the peaks corresponding to the other two cannabinoids, CBD and CBN, were much lower than that of THC, and in some of the samples, the concentration of CBN was below its LOQ (see Table 1). Consequently, THC was the only compound used for quantitative studies.

The percentages of THC in the plants were estimated from the peak areas measured for this compound in the collected extracts and the calibration equation of Table 1, taking into account the dilution factor. As conditions for the extraction were those proposed in a previously validated method [18], it was assumed that extraction of cannabinoids from the plants was quantitative. The results obtained for plant extracts after applying different dilution factors (1:100–1:200) are listed in Table 2. 

As observed in this table, the percentages of THC found for samples exposed to ambient conditions were <1%. The values obtained for three independent assays for one of the samples (M1) were 0.4%, 0.4%, and 0.8%. This variability (relative standard deviation, RDS = 23%) can be explained by the heterogeneity of the sample. It has to be noted that all samples were processed as they were expected to be consumed by users, that is, without being homogenized with lab equipment such as mortars or mills. The consecutive analysis of three aliquots of the same extract led to peak areas of THC with a relative standard deviation of 4%.In samples dried at 50 °C, the percentages of THC slightly increased (up to 4.8%) most probably due to the loss of humidity. These values are about the same order as those reported by other authors [17,18,19]. As for the samples exposed to ambient conditions, the precision was evaluated by performing three independent analyses of the same sample (M1), with the resulting relative standard deviation (RSD) of 12%. In another set of experiments, one of the extracts was spiked with standard solutions of the analytes (added concentration, 50 ng/mL). Then, the increments on the peak areas were used to estimate the added concentration from the calibration equations of Table 1. The calculated concentration to added concentration ratios were used to calculate the recoveries, and the mean values were 91%, 88%, and 128% for CBN, CBD, and THC, respectively. It was concluded that the analyte responses were not substantially affected by the matrix and, therefore, the values presented in Table 1 were valid for the analysis of cannabis plant extracts. 

### 2.3. Analysis of Residues of Cannabis on Surfaces

#### 2.3.1. Collection and Extraction Procedure

**Figure 1:**
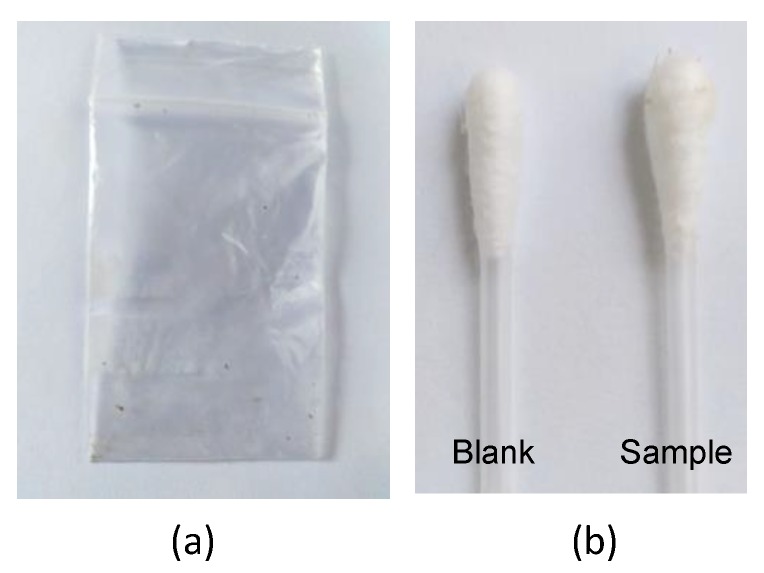
Images obtained in the studies for the analysis of residues of cannabis: (**a**) a plastic bag after the removal of the plant; (**b**) swabs after collecting the residue; and an unused swab (blank).

In order to develop a protocol for the analysis of contact traces of cannabis, different studies were carried out using plastic as a model surface, more specifically, polyethylene bags (6 × 4 cm). The bags were previously put into contact with cannabis by placing about 1.0 g of plant inside them and pressing it. Then, the bags were emptied by shaking them repeatedly, so that most parts of the plants were removed (only small particles could be visually detected). Next, the inner surface of the bag was wiped with a cotton swab in order to collect possible residues of the plant. The amount of residue collected was calculated by the difference of mass of the swabs before and after the wiping step. As an illustrative example, Figure 1 displays images of one of the bags (a) and the swab obtained after wiping the bag as well as an unused swab (blank) (b). The mass of the residues collected during the study ranged from 0.08 to 0.87 mg. It has to be noted that, unlike other procedures, dry swabs were used to collect the traces of cannabis in order to avoid error during weight operations due to evaporation of the solvent. 

Next, the cotton tips of the swabs were introduced into 2-mL glass vials, and after adding 1 mL of the extracting solvent (the cotton tip of the swab was completely soaked), the vials were introduced into an ultrasonic bath for 15 min. Finally, the liquid phase was removed and filtered for further processing. 

As explained above, the high sensitivity attainable by IT-SPME-nanoLC allowed the detection and quantification of the main cannabinoids in only 10 mg of plants, making the dilution of the collected extracts necessary. However, in the analysis of traces of cannabinoids, much lower amounts of samples are expected to be available. Therefore, dilution factors as low as possible should be applied. In an attempt to eliminate intermediate dilutions and since water-rich media are necessary for IT-SPME, water and different water–methanol mixtures were tested for the extraction of THC from the residues of cannabis collected on the swabs. The results were compared with those obtained by using methanol and chloroform (9:1, *v*/*v*). 

No THC was detected in the chromatograms obtained when using water for extraction. According to previous works, a water-methanol mixture (5:1, *v*/*v*) is suitable for IT-SPME with a PDMS-based coating, such as that used in the present study [20]. However, with this solvent, the extraction of THC was also unacceptably low, which can be explained by its high hydrophobicity (log k_octanol/water_ = 6.97) [21]. Much better results (higher peak areas for the analytes) were observed when the extraction was carried out with methanol, followed by the dilution of the extracts with water. On the other hand, no significant increments of the peak areas were observed when methanol:chloroform was used for extraction. For simplicity, methanol was selected for extraction in further assays. 

**Figure 2:**
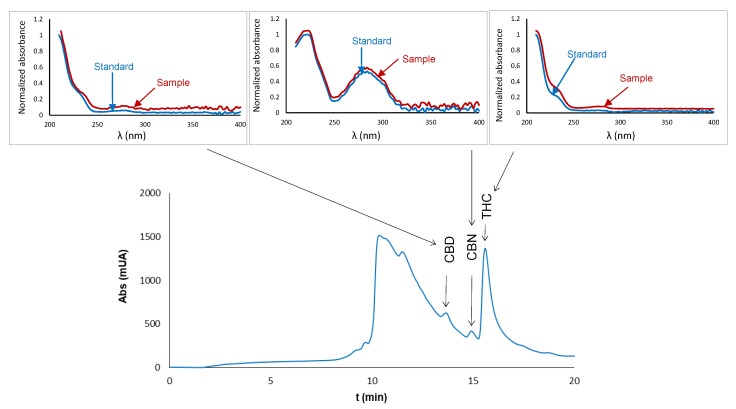
Chromatogram obtained in the analysis of residues of cannabis (sample M1, 0.82 mg), and comparison of the spectra obtained for the peaks of the suspected cannabidiol (CBD), cannabinol (CBN), and THC and those of standard solutions. The methanolic extract collected after processing the swab was diluted 1:5 with ultrapure water before the IT-SPME-nanoLC step. Conditions used for IT-SPME and chromatographic analysis were identical to those indicated in Section 4.3 and Section 4.2, respectively.

As an example, Figure 2 shows the chromatograms obtained for one of the samples (M1). The amount of sample collected with the swab was 0.82 mg, and the methanolic extract was diluted with ultrapure water, with a methanol:water proportion of 1:5 (*v*/*v*). This figure also shows the good concordance between the UV spectra recorded for the peaks of the suspected analytes and those obtained for standard solutions of the analytes. Therefore, the presence of cannabis in the collected trace sample could be properly confirmed. 

#### 2.3.2. Type of Surface

**Figure 3:**
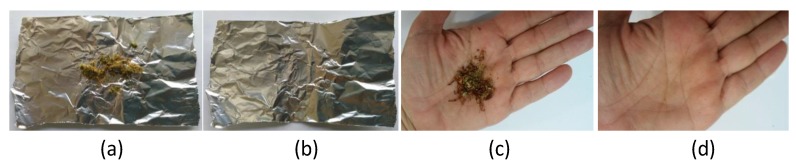
Images obtained in the studies for the analysis of residues of cannabis on: aluminum foil in contact with cannabis (**a**) and after the removal of the plant (**b**); hand of a volunteer in contact with cannabis (**c**); and after the removal of the plant (**d**).

The proposed procedure was applied to detect residues of cannabis on other surfaces, namely, aluminum foil (7 × 10 cm), office paper (5 × 5 cm), a piece of cotton cloth (2 × 2 cm), and skin (hand). In the assays with the office paper, a piece of cloth and hand small particles of cannabis were visually detected after the removal of the drug; no traces were detected by naked eye on the aluminum, which was the less porous material (see Figure 3).

Possible residues of cannabis were collected with swabs, and the swabs were treated with 1 mL of methanol as described above. For comparison purposes, a piece of the cotton fabric (2 × 2 cm) was also directly immersed in 2 mL of methanol, and the extract was treated the same as the solutions obtained for the swabs. 

**Figure 4:**
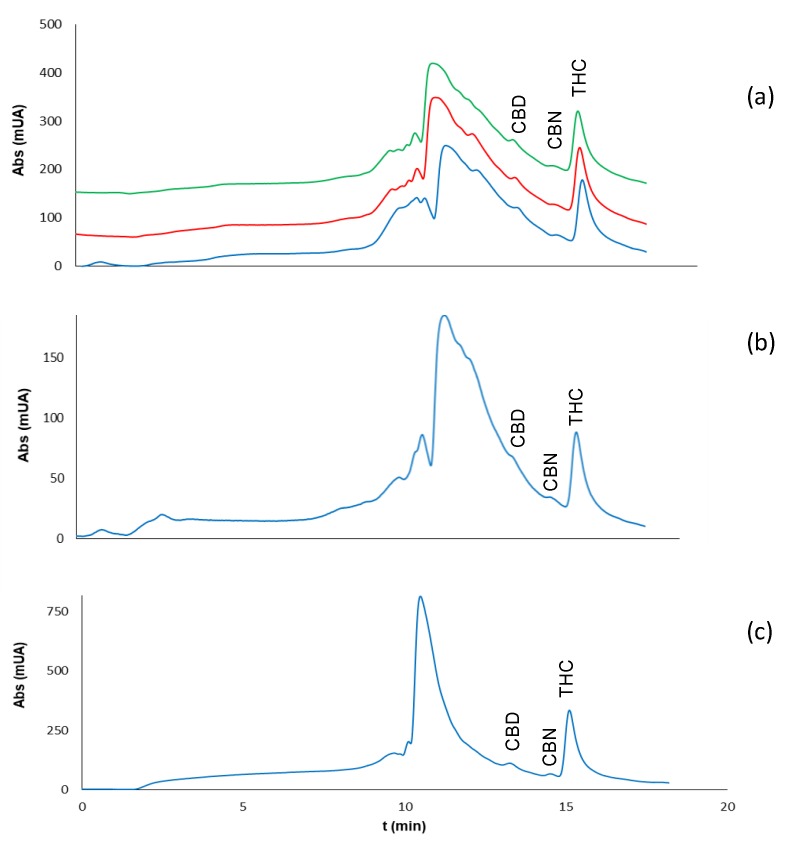
Chromatograms obtained for the analysis of residues of cannabis (sample M1) in: (**a**) aluminum foil, (**b**) cellulose paper, and (**c**) and skin. The sample amounts collected with the swabs were 0.27, 0.10, and 0.25 mg in (**a**–**c**), respectively; the extracts were diluted 1:20 with ultrapure water before the IT-SPME–nanoLC step in (**a**,**b**) and 1:5 in (**c**). Conditions for sampling and extraction were identical to those indicated in Section 2.3.1; conditions used for IT-SPME and chromatographic analysis are indicated in Section 4.3 and Section 4.2, respectively.

The amounts of samples collected were of about the same order as those collected in the assays with plastic bags, but the amount of cannabis that remained bound to the piece of cloth was much higher (4.10 mg). The three cannabinoids could be detected and identified, although the concentration of CBN in some of the samples was below its LOQ. Examples of the chromatograms obtained are given in Figure 4. 

#### 2.3.3. Quantitation of THC

The percentage of THC in the collected residues was established from the peak areas of the chromatograms obtained. The dilution factor was selected according to the amount of residue collected with the swabs; dilution factors of 1:20–1:100 were adequate in most of the samples to adjust the concentrations of THC to the linear concentration interval (Table 1). However, due to the high amount of cannabis collected in the assay with the piece of cloth, a dilution factor of 1:250 had to be applied. 

The results obtained in quantitative assays are summarized in Table 3. As observed in this table, the concentrations of THC measured were in the 41–99 µg/mL concentration interval. These concentrations correspond to percentages of THC in the residues ranging from 0.6% to 2.8%. These values were similar or slightly higher than those found for the plants, which indicates that the parts of the plant with a higher tendency to adhere to surfaces are those with higher THC contents. 

The intraday precision of the method was estimated for the entire procedure from three independent consecutive analyses of the same cannabis sample (M1). The RSD obtained was 17%. The interday precision was calculated from the analysis of residues of the sample M1 on three different days, resulting in RSD 27% (*n* = 3). The reproducibility was also tested for the consecutive injection of aliquots of the same extract. The RSD values obtained were ≤18% for bags, 4% for aluminum foil (see also Figure 3a), 6% for cellulose paper, 21% for cloth, and 7% for skin. As for the assays with plants, a slight increment in the percentage of THC was found for samples previously dried at 50 °C. 

#### 2.3.4. Selectivity

**Figure 5:**
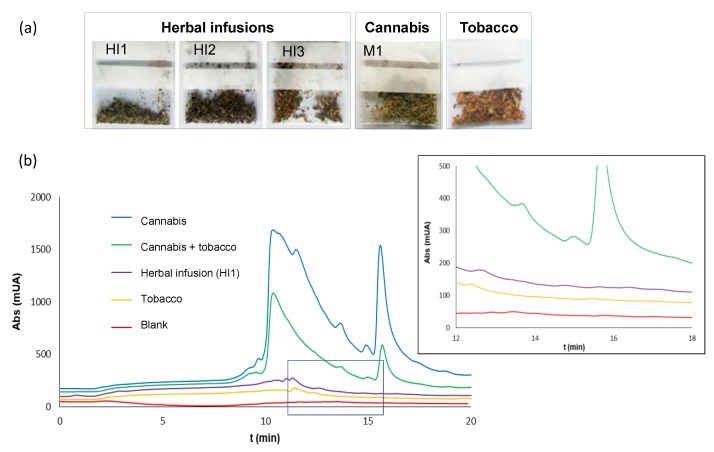
(**a**) Images of the bags with the different products used in the selectivity study: herbal infusions (HI1–HI3), tobacco, and cannabis (M1). (**b**) Chromatograms obtained for the analysis of residues collected from some of the bags (**a**) after the removal of the products: cannabis (M1), tobacco, a mixture of M1 and tobacco (1:4, m/m), herbal infusion (HI1), and a blank. The extracts were diluted 1:20 with ultrapure water before being processed by IT-SPME-nanoLC. Conditions for sampling and extraction were identical to those indicated in Section 2.3.1; conditions used for IT-SPME and chromatographic analysis were identical to those indicated in Section 4.3 and Section 4.2, respectively.

The proposed method was applied to other kinds of samples in order to evaluate the selectivity; the products tested were three herbal infusions (HI1–HI3) and tobacco (see Figure 5a). A mixture of tobacco:cannabis in a proportion of masses of 4:1 was also tested. This study was carried out with plastic bags. 

The chromatograms obtained for all the herbal infusions and tobacco tested were free of peaks in the region where CND, CBN, and THC eluted. Examples of the chromatograms obtained are depicted in Figure 5b. As observed in this figure, in the tobacco and cannabis mixture, the three analytes were detected, with their respective peak areas being about a quarter of those found when the assay was carried out with cannabis (chromatogram also shown in Figure 5b). 

In view of these results, it was concluded that the proposed approach was suitable for the differentiation of residues of cannabis from noncannabinoid plants. 

## 3. Discussion

The analysis of cannabis has been undertaken from different perspectives such as the characterization of the varieties of plants or the quantification of the active components [17,18,19], typically using LC-DAD or LC-MS(/MS). Chromatographic techniques have been also used for confirmatory analysis of samples that give positive results in screening tests for drugs, for example, in colorimetric tests [22]. Those methods are not suitable for the analysis of contact traces of cannabis because the amounts of sample required are much higher, typically 100–300 mg [15,16,17]. Only a few procedures have been described for the detection and quantification of THC in surfaces so far and have been generally addressed to the evaluation of the exposure to cannabis on work surfaces [9,10,11,12]. Those methods allow the detection and quantification of THC (LOQ, 5 ng/mL) with analyte detectability similar to that of the present approach, but they involve wiping larger areas (which may not always be possible) and multiple extraction/evaporation of the extracts (3 × 8 mL of methanol followed by evaporation to dryness). 

In the present study, we have illustrated the potential of IT-SPME coupled to nanoLC for the detection and characterization of contact traces of complex materials, such as cannabis plants. For the first time, it has been demonstrated that IT-SPME-nanoLC can be used for the analysis of extracts obtained for such samples with adequate selectivity. The system stability was also suitable; more than 100 extracts were analysed by the proposed method with the same capillary without observing deterioration in its retention properties or in the system background pressure. The proposed approach uses green extraction techniques, such as ultrasound assisted extraction, and only 1 mL of methanol per sample is necessary. It has been applied to the detection and quantification of amounts 1–2 µg of THC from areas typically lower than 50 cm^2^, but lower amounts could be detected. As an example, considering the instrumental detection limit (2 ng/mL), a percentage of THC of 1% in the sample, and a dilution factor 1:20, the method could detect THC in residues of cannabis of only 4 µg.

Throughout our study, we observed that most parts of the residues were collected with swabs. However, in more porous surfaces, incomplete removal of the sample could occur, and thus, the amount of residue collected would be affected by parameters such as pressure or time of sampling. In such cases, the proposed procedure could not be applied to estimate the mass of residue, although the positive identification of cannabis would be still possible. If required for a more complete characterization of the sample, the proposed approach could be applied to the quantification of CBD and CBN by processing the undiluted extracts.

Because of the low amount of sample necessary, the protocol can be considered minimally invasive. Moreover, as only 10 µL of diluted extracts is necessary, most part of the original extract can be stored and used in further studies, if required. The concordance of the percentages of THC found in the study with plants (Table 2) and with residues of the same plants (Table 3) confirms the reliability of the proposed approach.

In summary, this work illustrates the potential utility of new integrated techniques such as IT-SPME coupled on-line to nanoLC for contact trace analysis of complex samples [23].

## 4. Materials and Methods

### 4.1. Chemicals and Solutions

All reagents were of analytical grade. Standards of Δ^9^-trans-tetrahidrocannabinol, cannabidiol, and cannabinol (solutions in methanol) were obtained from Sigma-Aldrich (St. Louis, MO, USA). Acetonitrile, chloroform, and methanol were of HPLC grade (VWR, Radnor, PA, USA).

Intermediate stock standard solutions of the analytes (1 mg/mL) were prepared by diluting the commercial standards with acetonitrile and kept at −20 °C until use. Working solutions were prepared by diluting the stock solutions with ultrapure water.

### 4.2. Chromatographic Conditions

Chromatographic analyses were performed using an Agilent 1260 Infinity nanoLC chromatograph equipped with a quaternary nanopump, a six-port microscale manual injector (Rheodyne, Rohnert Park, CA, USA), and a UV-Vis diode array detector with an 80-nL nanoflow cell (Agilent, Waldbronn, Germany). The detector was coupled to a data system (Agilent, ChemStation) for data acquisition and treatment. The analytical signal was recorded between 190 and 400 nm and monitored at 210 nm. A Zorbax 300SB C18 (50 × 0.075 mm id, 3.5 µm particle size) column (Agilent) was used for separation.

The mobile phase was a mixture of water and acetonitrile in gradient elution mode. The percentage of acetonitrile in the mobile phase was linearly increased from 55% at 0–5 min to 75% at 10 min, which was then kept constant until 12 min; finally, the acetonitrile percentage was linearly decreased to reach a percentage of 55% at 15 min and remained constant until the end of the run. The flow rate was 0.5 µL/min. Solvents were filtered through 0.22-µm nylon membranes before use (Teknokroma, Barcelona, Spain).

### 4.3. IT-SPME Conditions

For the IT-SPME a segment of a TBR-5 column (95% polydimethyl siloxane, 5% polydiphenylsiloxane), 15 cm length, 0.320 mm id, and 3 µm coating thickness (Teknokroma, Barcelona, Spain), was used. For connecting the extractive capillary to the valve, a 2.5-cm sleeve of 1/6 i.n. polyether ether ketone (PEEK) tubing (1/6 i.n. PEEK nuts and ferrules (Teknokroma)) was used.

The volume of sample loaded into the IT-SPME device was 10 μL.

### 4.4. Analysis of Cannabis Plants

Four cannabis samples (M1–M4) collected from different plants within two to three months before the study were used. The samples, intended for self-consumption, were voluntarily donated by users who were previously informed of the aim of the study. The samples were stored in plastic bags in the dark until use.

For the extraction of cannabinoids, accurately weighted portions of the samples (10 mg) were placed in 10-mL glass vials, and then 3 mL of a mixture of methanol and chloroform (9:1, *v*/*v*) was added. The vials were closed with a screw cap and placed into an ultrasonic bath (300 W, 40 kHz, Sonitech, Guarnizo, Spain) for 15 min. Next, a portion of about 1 mL of the extraction solvent was removed and filtered through 0.22-µm nylon membranes (Teknokroma). Unless otherwise stated, the extracts were properly diluted with ultrapure water and processed by IT-SPEM-nanoLC. Each sample was assayed in duplicate.

### 4.5. Analysis of Traces of Cannabis on Surfaces

The presence of traces of cannabis on the surface of different types of materials was evaluated: plastic (polyethylene), cellulose office paper, aluminum foam, cotton cloth, and hand skin. For this purpose, the items were put into contact with portions of cannabis of about 1 g by exerting pressure with hands, and then the plant was removed by shaking the item repeatedly. Next, the surface of the item was wiped with dry swabs for 20–30 s in different directions in order to collect possible traces of cannabis; the surface areas wiped were of about 20–50 cm^2^. The swabs were accurately weighted before and after sampling, so the amount of sample collected was calculated as the difference of masses. After sample collecting, the swabs were placed in glass vials and the target compounds were extracted in 1 mL of methanol (unless otherwise stated) in an ultrasonic bath for 15 min. The extracts were properly filtered and diluted with ultrapure water and then processed by IT-SPEM-nanoLC. Unless otherwise stated, each extract was processed in duplicate.

Cotton swabs purchased from local supermarkets were used for collecting the samples.

For the selectivity studies, three different herbal infusions—lime-orange (HI1), pennyroyal (HI2), and valerian (HI3)—were used. These products were purchased from local supermarkets.

## Figures and Tables

**Table 1:** Quantitative performance of the proposed method (values established from aqueous standard solutions of the target compounds processed by in-tube solid-phase microextraction coupled on-line to liquid chromatography (IT-SPME-nanoLC)).

Compound	Linearity ^1^, y = ax + b			n	LOD (ng/mL)	LOQ (ng/mL)
	a ± s_a_ (mL/ng)	b ± s_b_	r^2^			
THC	24 ± 1	−270 ± 80	0.98	7	2	8
CBD	28 ± 2	−300 ± 100	0.990	7	5	15
CBN	7.8 ± 0.5	−10 ± 30	0.990	6	5	15

^1^ Tested up to concentrations of 100 ng/mL of each compound.

**Table 2:** Percentages of Δ^9^-tetrahydrocannabinol (THC) in cannabis plants stored at ambient conditions and at 50 °C.

Storage Conditions	Percentage of THC (%) ^1^			
	M1	M2	M3	M4
Ambient	0.4, 0.8, 0.8	0.4, 0.5	0.4, 0.8	0.6, 0.7
50 °C	1.0, 1.0, 0.8	3.2, 2.8	4.8, 4.7	3.2, 2.6

^1^ Values obtained from three independent assays for sample M1 and for two replicates of the same plant extract in samples M2–M4.

**Table 3:** Mean concentrations of THC found in the analysis with residues of THC (*n* = 3), and their equivalence in amount of THC collected and percentage of THC in the samples.

Item	Cannabis Sample	Amount of Sample Collected (mg)	Dilution Factor	Concentration of THC Measured (ng/mL)	Estimated Amount of THC Collected (µg)	Percentage of THC in the Sample (%)
Plastic bag	M1	0.82	1:100	79	8.2	1.0
Plastic bag	M2	0.42	1:50	78	3.8	0.9
Plastic bag	M3	0.87	1:400	61	24.4	2.8
Plastic bag	M4	0.38	1:100	99	9.9	2.6
Plastic bag	Mixture of plants	0.10	1:20	58	1.2	1.2
Plastic bag	Mixture of plants	0.08	1:100	41	4.1	5.1
Aluminum foil	M1	0.27	1:20	82	1.6	0.6
Office paper	M1	0.10	1:20	75	1.5	1.5
Piece of cotton cloth	M1	4.28	1:250	71	34.2	0.4
Skin	M1	0.20	1:20	94	1.9	0.9
